# Identification of Small Molecules Inhibiting Cardiomyocyte Necrosis and Apoptosis by Autophagy Induction and Metabolism Reprogramming

**DOI:** 10.3390/cells11030474

**Published:** 2022-01-29

**Authors:** Dawei Liu, Félix Peyre, Yahir Alberto Loissell-Baltazar, Delphine Courilleau, Sandra Lacas-Gervais, Valérie Nicolas, Eric Jacquet, Svetlana Dokudovskaya, Frédéric Taran, Jean-Christophe Cintrat, Catherine Brenner

**Affiliations:** 1 Centre National de Recherche Scientifique (CNRS), Institut Gustave Roussy, Aspects Métaboliques et Systémiques de l’Oncogénèse pour de Nouvelles Approches Thérapeutiques, Université Paris-Saclay, 94805 Villejuif, France; Dawei.liu@gmail.com (D.L.); felix.peyre@gmail.com (F.P.); yahiralberto.LOISSELL-BALTAZAR@gustaveroussy.fr (Y.A.L.-B.); svetlana.dokudovskaya@gustaveroussy.fr (S.D.); 2 Inserm, Centre National de Recherche Scientifique (CNRS), Ingénierie et Plateformes au Service de l’Innovation Thérapeutique, Université Paris-Saclay, 92296 Châtenay-Malabry, France; delphine.courilleau@u-psud.fr (D.C.); valerie.nicolas@universite-paris-saclay.fr (V.N.); 3 Centre Commun de Microscopie Appliquée, CCMA, Université Côte d’Azur, 06103 Nice, France; Sandra.LACAS-GERVAIS@univ-cotedazur.fr; 4 Institut de Chimie des Substances Naturelles, Université Paris-Saclay, CNRS, 91190 Gif-sur-Yvette, France; Eric.JACQUET@cnrs.fr; 5 Département Médicaments et Technologies pour la Santé (DMTS), Université Paris Saclay, CEA, INRAE, SCBM, 91191 Gif-sur-Yvette, France; frederic.taran@cea.fr (F.T.); jean-christophe.cintrat@cea.fr (J.-C.C.)

**Keywords:** apoptosis, autophagy, cardioprotection, cardiotoxicity, mitochondrion, screening

## Abstract

Improvement of anticancer treatments is associated with increased survival of cancer patients at risk of cardiac disease. Therefore, there is an urgent need for new therapeutic molecules capable of preventing acute and long-term cardiotoxicity. Here, using commercial and home-made chemolibraries, we performed a robust phenotypic high-throughput screening in rat cardiomyoblast cell line H9c2, searching for small molecules capable of inhibiting cell death. A screen of 1600 compounds identified six molecules effective in preventing necrosis and apoptosis induced by H_2_O_2_ and camptothecin in H9c2 cells and in rat neonatal ventricular myocytes. In cells treated with these molecules, we systematically evaluated the expression of BCL-2 family members, autophagy progression, mitochondrial network structure, regulation of mitochondrial fusion/fission, reactive oxygen species, and ATP production. We found that these compounds affect autophagy induction to prevent cardiac cell death and can be promising cardioprotective drugs during chemotherapy.

## 1. Introduction

One of the major problems in anticancer treatments is the management of toxicity that affects cardiac cells and leads to cardiac dysfunction and cardiomyopathy in many surviving patients. The number of patients at risk for cardiovascular diseases increases in correlation with the improvement of survival for most cancers resulting in higher cardiovascular morbidity and mortality [1,2].

Acute cardiac damages can be induced by tissue irradiation and chemotherapy, especially upon treatment with tyrosine kinase inhibitors and anthracyclines (doxorubicin and epirubicin), as often observed in childhood cancer survivors [1,2,3,4,5,6]. Cardiotoxicity can also develop in Her2-positive breast and stomach cancer patients treated with trastuzumab and other Her2-targeted drugs since Her2 is expressed not only in tumors but also in cardiomyocytes. Depending on the anticancer agent and patient comorbidities, cardiotoxicity mechanisms can involve DNA damage, endoplasmic reticulum stress, mitochondrial dysfunction, reactive oxygen species (ROS) production, bioenergetic metabolism failure, apoptosis, and necrosis [6,7]. Of note, necrosis and apoptosis in the heart differ in terms of triggering stimuli, biochemical effectors, and sequence of the events leading to cell death [6,7]. For example, plasma membrane permeabilization occurs early in necrosis and lately in apoptosis. Therefore, there is an urgent need to develop new cardioprotective molecules capable of preventing cardiotoxicity in cancer patients.

Here, in perspective to find novel cardioprotective drug candidates, we performed a phenotypic high-throughput screening using a rat cardiomyoblast cell line, H9c2, and tested commercial and home-made library of 1600 molecules searching for compounds capable of inhibiting both apoptosis and necrosis. We used camptothecin, a potent apoptosis inducer that acts both as the DNA-intercalating agent and topoisomerase I inhibitor [8] and H_2_O_2_, which causes oxidative damage and induces both necrosis and apoptosis [9]. We identified six molecules that could be used to maintain cardiomyocyte viability preventively during treatment with H_2_O_2_ or camptothecin and further characterized their cellular and molecular effects in rat primary neonatal cardiomyocytes (RNVCs). To be effective, all molecules require autophagy regulators ATG5 and BECLIN-1 proteins but have differential abilities to regulate cell death, autophagy, and mitochondrial structure. Overall, these compounds are promising cardioprotective drugs to be used in the course of chemotherapy and should be further tested during preclinical studies.

## 2. Material and Methods

### 2.1. Phenotypic High Throughput Screening

#### 2.1.1. Chemical Libraries

Compounds obtained from Prestwick library (1200 molecules) and CEA SCBM library (400 molecules) were dissolved at 10 mM in 100% DMSO to prepare stock solutions. The distribution of compounds into 96 well plates was made with a Biomek Single Bridge 96 liquid handler (Beckman Coulter, Brea, CA, USA). 

#### 2.1.2. Cellular Treatments

H9c2 cells (ATCC 30-2002™) were cultivated in Dulbecco’s Modified Eagle’s Medium (DMEM) complemented with Fetal Bovine Serum 10% (BioWhittaker, Walkersville, MD, USA) and penicillin-streptomycin mixture (Gibco, Waltham, MA, USA). H9c2 cells were seeded in 96 well plates (5.000 cells/well), let adhere for 48 h, and treated with compounds at 10 µM for 2 h at 37 °C. Compounds were removed and replaced with a culture medium containing either 10 µM camptothecin (Sigma, St. Louis, MO, USA) 24 h to induce apoptosis or 300 µM H_2_O_2_ (Sigma, St. Louis, MO, USA) 2 h to induce necrosis, and 0.1% DMSO in culture medium was used as a negative control.

#### 2.1.3. Viability Measurement and Hit Selection

The percentage of viable cells was evaluated by methylene blue staining [10]. After treatment, cells were washed two times with PBS and fixed with ethanol for 30 min at room temperature. Ethanol was removed, and plates were left to dry overnight; cells were stained with 0.1 g/L methylene blue for 5 min, washed three times with water, and resuspended in 100 mM HCl. Absorbance was measured at 665 nm (Envision spectrofluorimeter, Perkin Elmer, Waltham, MA, USA). Results were normalized with negative control, and hits were selected if the absorbance value was higher than the mean cell death value plus 3 standard deviations (SD).

### 2.2. Neonatal Cardiomyocyte Isolation

Rat neonatal cardiomyocytes (RNVCs) were isolated as previously described [11]. Briefly, RNVCs were isolated from Wistar newborn rat hearts, and cells were cultured in Minimum Essential Medium (MEM) containing 1.2 mM Ca^2+^, 2.5% fetal bovine serum (FBS), 1% penicillin-streptomycin, and 2% HEPES (pH 7.6) and plated on culture dishes, coated with 10 μg/mL laminin. RNVCs were left to adhere for 2 h in a 95% O_2_, 5% CO_2_ at 37 °C before the medium change.

### 2.3. LDH Release Assay

A colorimetric assay was used to measure lactate dehydrogenase (LDH), a cytosolic enzyme released upon plasma membrane permeabilization, and to evaluate cell viability. Assay from Promega was performed using cell culture supernatants obtained from H9c2 cells or RNVCs, and LB was used as a positive control of total cell lysis. LDH release was measured at 490 nm (Infinite spectrofluorimeter, Tecan, Zurich, Switzerland).

### 2.4. Plasma Cell Membrane Permeabilization Assay

Propidium iodide (Sigma, St. Louis, MO, USA), a fluorescent impermeable DNA marker, was used to measure plasma membrane integrity. Propidium iodide at 10 µM was added in the culture medium, and fluorescence reading was performed (λex: 530 nm; λem: 620 nm) using TECAN infinite spectrofluorimeter (Tecan, Zurich, Switzerland), commercial lysis buffer (LB) was used as a control.

### 2.5. Plasmid Transfection

Then, 4 × 10^5^ neonatal cardiomyocytes were plated overnight on 35 mm culture dishes coated with 10 µg/mL laminin, and 24 h later, cells were transiently transfected with 1 µg plasmid coding for GFP-LC3 (generous gift from Dr. J.L. Perfettini, INSERM U1030, Gustave Roussy, Villejuif, France) by using 2.5 µL Lipofectamine^®^ 2000 (Thermo Fisher, Waltham, MA, USA) for 48 h. Fluorescence was detected with a confocal microscope (SP5 Leica). Images were analyzed with Image J (Wayne Rasband, NIH, Bethesda, MD, USA).

### 2.6. Mitochondrial Network Analysis by Confocal Microscopy and Transmission Electron Microscopy

#### 2.6.1. Confocal Microscopy

4 × 10^5^ RNVCs were plated overnight on 35 mm culture dishes coated with 10 µg/mL laminin, and 24 h later, cells were treated for 6 h with 1 µM or 10 µM of different compounds. Cells were incubated with Mitotracker Red 580 at 200 nM for 20 min at 37 °C, then with 4 μM calcein (Life Technologies, Carlsbad, CA, USA) for 10 min at 37 °C. Z stack images were acquired with a Leica (TCS SP8 gSTED) inverted confocal laser scanning microscope (Mannheim, Germany) equipped with a WLL Laser (495 nm excitation wavelength for calcein and 580 nm for Mitotracker Red 580). Green fluorescence emission was detected with 505–550 nm wide emission slits and 585–700 nm wide emission slits for the red signal under a sequential mode. The pinhole was set at 1.0 Airy unit, and 12-bit numerical images were done with the Leica Application Suite X software (Version 3.5.5; Leica, Wetzlar, Germany).

Mitochondrial network and cell volume 3D model were reconstructed by using the IMARIS software 9.7 version (Bitplane Company, Zurich, Switzerland); consequently, cell volume, mitochondria number, and volume were analyzed using the volume and surface rendering processes.

#### 2.6.2. Transmission Electron Microscopy

For ultrastructural analysis, cells were fixed in 1.6% glutaraldehyde in 0.1 M phosphate buffer, pH 7.3, washed in 0.1 M cacodylate buffer, fixed for 1 h in 1% osmium tetroxide, and 1% potassium ferrocyanide in 0.1 M cacodylate buffer to enhance the staining of membranes [12]. Cells were washed in distilled water, dehydrated in alcohol, and embedded in epoxy resin. Contrasted ultrathin sections (70 nm) were analyzed under a JEOL 1400 transmission electron microscope equipped with a Morada Olympus CCD camera.

### 2.7. ROS Detection in RNVCs

A total of 50 μg of MitoSOX mitochondrial superoxide indicator (MitoSOX™, Thermo Fisher, Waltham, MA, USA) was dissolved in 13 μL of DMSO to make 5 mM MitoSOX™ stock solution, which was further diluted in PBS to make a 5 μM MitoSOX working solution. RNVCs were treated with either 0.1% DMSO or 3 μM rapamycin, or 1 μM solutions of digitoxigenin, digoxin, SG6163F VP331, LOPA87, or minaprine in cell culture medium for 6 h. After treatments, cells were washed 2 times with PBS at 37 °C, incubated with 5 μM MitoSOX for 10 min at 37 °C, and gently washed three times with warm PBS. The nuclear fluorescence was deleted, and mitochondrial fluorescent intensity was measured by using ImageJ software.

### 2.8. Real-Time Bioenergetic Profile Analysis in H9c2 Cardiomyocytes

The XFe96 Extracellular Flux Analyzer (Seahorse Biosciences, North Billerica, MA, USA) was used to measure cellular bioenergetic function. H9c2 cells were seeded at 20,000 cells per well in XFe96 cell culture microplates; all the pre-treatments were performed with a serum-free cell culture medium. The Agilent Seahorse XF Glycolysis Stress Test Kit (Agilent, Santa Clara, CA, USA) was used to measure glycolytic function by quantification of the extracellular acidification rate (ECAR) followed by 3 sequential injections of 10 mM glucose, 2 µM oligomycin, and 50 mM 2-deoxy-D-glucose. The oxygen consumption rate (OCR) was measured with Seahorse XF Cell Mito Stress Test Kit (Agilent, Santa Clara, CA, USA). The built-in injection ports on XF sensor cartridges were used to add modulators of respiration into cells during the assay to reveal the key parameters of mitochondrial function. Then, 2 µM oligomycin was injected first, followed by the addition of 1 µM carbonyl cyanide-4 (trifluoromethoxy) phenylhydrazone (FCCP). Finally, 0.5 µM antimycin A was injected to stop mitochondrial respiration. The oxidation of exogenous fatty acids was measured using the XF Palmitate-BSA FAO Substrate kit (Agilent, Santa Clara, CA, USA) and the XF cell Mito Stress Test kit. Cells were grown in DMEM supplemented with 0.5 mM glucose, 1 mM GlutaMAX, 0.5 mM carnitine, and 1% fetal bovine serum. The FAO Assay Medium (111 mM NaCl, 4.7 mM KCl, 1.25 mM CaCl_2_, 2 mM MgSO_4_, 1.2 mM NaH_2_PO_4_, supplemented on the day of the assay with 2.5 mM glucose, 0.5 mM carnitine, and 5 mM HEPES pH 7.4), was kept at 37 °C. H9c2 cells were seeded at 20,000 cells per well in XF96 cell culture microplates; all the pre-treatments were performed with a serum-free cell culture medium. A total of 24 h prior to the assay, the growth medium was replaced with the substrate-limited medium, and 45 min prior to the assay, cells were washed two times with FAO Assay Medium; 150 μL/well FAO Assay Medium was added to the cells and incubated in a non-CO_2_ incubator for 30–45 min at 37 °C. The assay cartridge was loaded with XF Cell Mito Stress Test compounds (final concentrations: 2 µM oligomycin, 1 μM FCCP, and 0.5 μM antimycin A). Finally, 30 μL XF Palmitate-BSA FAO Substrate or BSA was added to the appropriate wells, then immediately inserted the XF Cell Culture Microplate into the XFe96 Analyzer for analysis.

### 2.9. SDS-PAGE and Western Blot

H9c2 cells and RNVCs were detached in LB containing 50 mM Tris pH 8.0, 150 mM NaCl, 1 mM EDTA, 0.5% deoxycholate, 1% Triton X 100, and 0.1% SDS. The cells were collected, placed on ice for 30 min and centrifuged at 2000× *g* for 20 min at 4 °C. The supernatant was transferred to a new tube and kept on ice. The protein concentration was determined by BCA assay. The protein samples were diluted with 2X Laemmli Sample Buffer (Sigma, St. Louis, MO, USA), incubated for 5 min at 95 °C, and loaded in 4–20% Tris-Glycine gel (Fisher Scientific, Waltham, MA, USA). Separated proteins were transferred onto PVDF membrane for 3 min at 2.5 V in Trans Blot Turbo System (BioRad, Hercules, CA, USA). The membrane was blocked with 5% milk in PBS/0.1% Tween and incubated overnight with an appropriate primary antibody in 5% milk in PBS/0.1% Tween at 4 °C. The membrane was washed 6 times × 5 min with PBS/0.1% Tween, incubated with a Horseradish Peroxidase-Conjugated secondary antibody for 1 h at room temperature, washed again with PBS/0.1% Tween, incubated with an ultra-sensitive enhanced chemiluminescent substrate for 5 min, and visualized with a gel imaging system (BioRad, Hercules, CA, USA). The following antibodies were used: anti-Mitofusin 1 (ab126575, Abcam, Waltham, MA, USA), anti-Mitofusin 2 (ab124773, Abcam, Waltham, MA, USA), BCL-2 (C-2) (sc-7382, Santa Cruz Biotechnology, Dallas, TX, USA), BAX (B-9) (sc-7480, Santa Cruz Biotechnology, Dallas, TX, USA), BCL-XL (2764, Cell Signaling, Danvers, MA, USA), LC3B (D11) (3868, Cell Signaling, Danvers, MA, USA), β-actin (C4) (sc-47778, Santa Cruz Biotechnology, Dallas, TX, USA), phospho-DRP1 (Ser616) (D9A1) (4494, Cell Signaling, Danvers, MA, USA), and DRP1 (611112, BD Biosciences, San Jose, CA, USA).

### 2.10. Statistical Analysis

Results are expressed as mean ± standard error (SD) or standard error to the mean (SEM). The Origin software and Graphpad Prism 6 were used for statistical analysis. Differences between 2 groups were analyzed by one-way ANOVA, and differences between groups of two genotypes were analyzed by two-way ANOVA, Sidak’s multiple comparisons. Statistical significance is indicated as follows: * *p* < 0.05, ** *p* < 0.01, *** *p* < 0.001, and **** *p* < 0.0001. 

## 3. Results

### 3.1. Identification of Cardiomyocyte Apoptosis and Necrosis Inhibitors by High Throughput Screening

**Figure 1:**
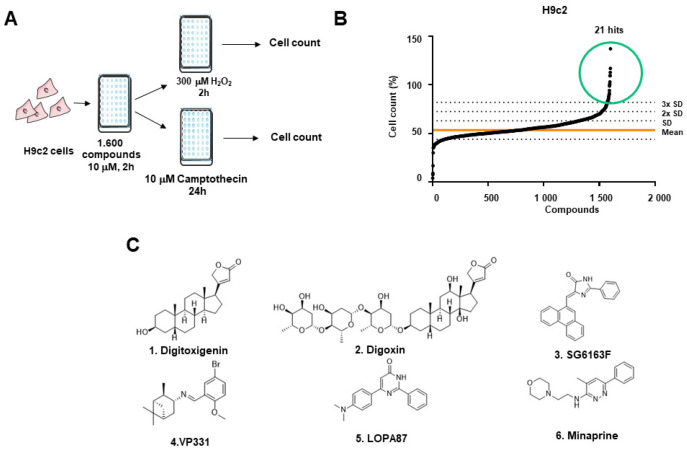
High-throughput screening for cardiac apoptosis and necrosis inhibitors. (**A**) Flow chart of the screening. Immortalized H9c2 cells were plated into 96-well plates, treated by 1600 compounds for 2 h, then by 10 μM camptothecin for 24 h or 300 μM H_2_O_2_ for 2 h. (**B**) Cell survival was determined by methylene blue staining, percentage of survived cells was calculated in comparison to 0.1% DMSO as the vehicle and used to rank the compounds. (**C**) Ranked list and chemical formula of 6 best hits selected from Prestwick (hits 1, 2, and 6) and SBM CEA libraries (hits 3, 4, and 5).

**Figure 2:**
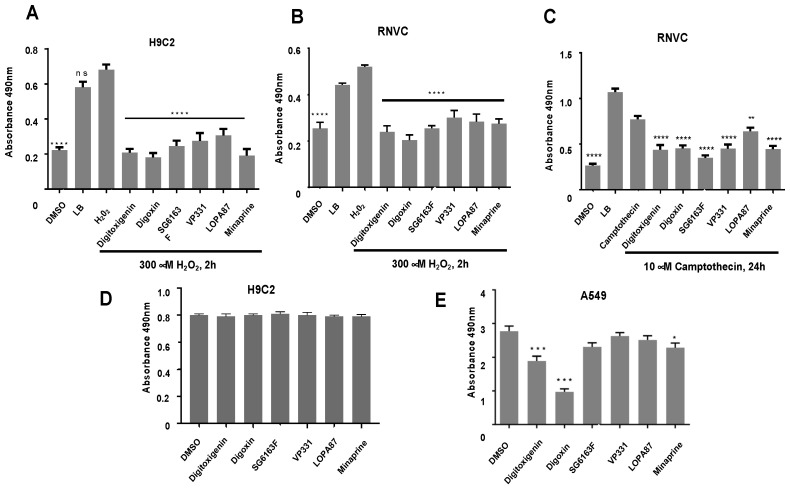
Comparative analysis of cell viability after treatment of various cells with selected compounds. Cell viability was evaluated by LDH release assay. H9c2 cells (**A**) or RNVCs (**B**,**C**) were first cultured with indicated compounds followed by treatment with 300 μM H_2_O_2_ for 2 h (**A**,**B**) or 10 μM camptothecin for 24 h (**C**). Evaluation of compound’s effect on cell growth in H9c2 cells (**D**) or lung carcinoma A549 cells (**E**). Cells were cultured in the presence of 10 μM compounds for 48 h and lysed with lysis buffer (LB) before LDH assay. Experiments were repeated three times. Data are presented as mean ± standard error to the mean (SEM) with one-way ANOVA, Sidak’s multiple comparisons test. *, *p* < 0.05, **, *p* < 0.01, ***, *p* < 0.001, ****, *p* < 0.0001 vs. 300 μM H_2_O_2_ (**A**), 10 μM Camptothecin (**C**), or 0.1% DMSO (vehicle) (**A**–**E**). ns, not significant.

To identify inhibitors of H_2_O_2_-induced necrosis and camptothecin-induced apoptosis in rat cardiomyoblast H9c2 cell line, a phenotypic high-throughput screening was performed with 1200 molecules from the commercial library Prestwick and 400 molecules from the home-made chemical library (Figure 1A). Our screen revealed 21 statistically significant hits (Figure 1B), of which we chose to investigate further six compounds that were most potent during cell death inhibition. Three of these six molecules (digitoxigenin, digoxin, and minaprine) belong to the Prestwick library, and three others are new chemical entities named SG6163F, VP331, and LOPA87 [13,14] (Figure 1C). Among the selected compounds, digitoxigenin and digoxin exhibited the best protection from cell death inducers, while minaprine was less powerful. The effect of these components was further confirmed in LDH release assay (Figure 2A) and propidium iodide staining (Supplementary Figure S1).

The efficacy of selected compounds to inhibit cell death after treatment with H_2_O_2_ or camptothecin was further confirmed on rat primary neonatal cardiomyocytes (RNVCs) using LDH assay and propidium iodide staining (Figure 2B,C and Figure S1). All six compounds efficiently inhibit both necrosis and apoptosis in RNVCs. To evaluate the longer-term effect of each compound, we cultured H9c2 cells and lung cancer cells A549 with the compounds for 24 h and 48 h but did not see any additional differences in comparison with the 6 h treatment. Therefore, in all following experiments, we used 6 h treatment time. Every compound, used as single agent, did not interfere with cell proliferation of H9c2 or A549 cells (Figure 2D,E), except digitoxigenin, digoxin, and minaprine, which significantly induced cell death of A549 cells (Figure 2E). 

**Figure 3:**
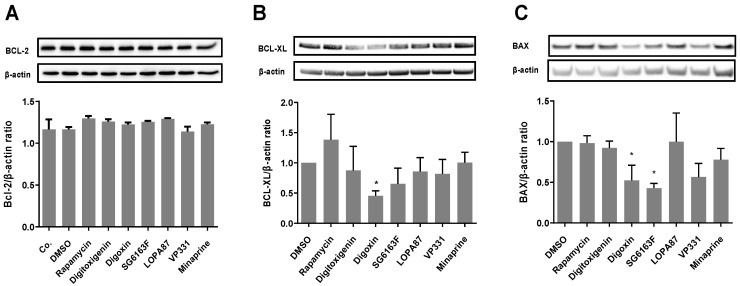
Effects of selected compounds on the expression of pro- and anti-apoptotic members of Bcl-2 family. Protein expression levels of BCL-2 (**A**), BXL-XL (**B**), and BAX (**C**) in RNVCs cultured for 6 h with indicated compounds. β-actin was used as a loading control. Co.—control of untreated cells. Representative Western blot images and quantification of three independent experiments are presented as mean ± SEM with one-way ANOVA, Sidak’s multiple comparisons test. *, *p* < 0.05 vs. DMSO.

To define cell death protective mechanisms of selected compounds, we first determined the protein expression level of anti-apoptotic B cell leukemia/lymphoma 2 family members BCL-2 and BCL-X and pro-apoptotic BAX (Figure 3) in RNVCs. The BCL-2 expression level was not changed after treatment with any compound (Figure 3A), whereas digoxin treatment decreased the expression of BCL-XL (Figure 3B). Digoxin and SG6163F decreased the expression of BAX (Figure 3C). Altogether, these results indicate the potential of the six compounds as necrosis and apoptosis inhibitors, with limited or no effect on BCL-2 family members’ expression.

### 3.2. SG6163F Influences Autophagy Induction via ATG5 and BECLIN-1

We further hypothesized that the effect of compounds on cell viability might be through activation autophagy, an evolutionarily conserved catabolic process that removes damaged or unnecessary cellular components [15]. In order to induce autophagy, we treated cells with rapamycin, an inhibitor of mTORC1 [16], but we did not observe significant effects on the expression of BCL-2 family members (Figure 3A–C).

**Figure 4:**
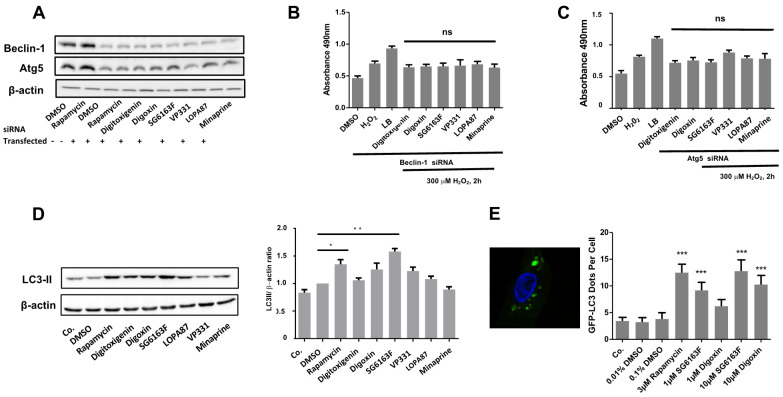
RNVCs cell death inhibition by compounds requires ATG5 and BECLIN-1. (**A**) RNVCs were transfected with pools of siRNAs targeting ATG5 or BECLIN-1, cultured for 48 h, and expression levels of both proteins were evaluated by Western blot. (**B**,**C**) Following BECLIN-1 (**B**) and ATG5 (**C**) siRNA transfection, LDH release was measured in RNVCs treated with 300 μM H_2_O_2_ for 2 h. Data are presented as mean ± SEM with one-way ANOVA, Sidak’s multiple comparisons test. ns.—not significant vs. H_2_O_2_ treated and siRNA transfected cells. (**D**) Protein level of LC3-II in RNVCs following treatment by compounds for 6 h was analyzed by Western blot, and the LC3II/b-actin ratio was determined in comparison to DMSO. Data are presented as mean ± SEM with one-way ANOVA, Sidak’s multiple comparisons test. *, *p*< 0.05, **, *p* < 0.01 vs. DMSO. (**E**) Redistribution of GFP-LC3. 24 h after transient transfection with a GFP-LC3 coding plasmid, cells were treated for 6 h with DMSO, rapamycin, and 1 and 10 μM of SG6163F and Digoxin. A representative cell is shown (left). The frequency of dots per cell (right) was quantified for 150 cells for each condition. Dots correspond to clear vacuolar distribution of GFP-LC3. Nuclei were stained by 0.5 μM Hoechst 33342. Data are presented as mean ± SEM with one-way ANOVA, Sidak’s multiple comparisons test. ***, *p* < 0.001 vs. DMSO. ns, not significant. +, transfection; -, no transfection.

We further checked whether ATG5 and BECLIN-1, two proteins necessary for autophagy induction [17,18], can play a role during the inhibition of apoptosis and necrosis by selected drugs. ATG5 and BECLIN-1 expression were downregulated by siRNAs transitory transfection in RNVCs for 24 h (Figure 4A). Cells were subsequently treated with six selected chemicals, incubated with H_2_O_2_ for 2 h, and cell death was analyzed by LDH assay (Figure 4B,C). In cells where ATG5 or BECLIN-1 expression was downregulated, all compounds lost their ability to protect RNVCs from H_2_O_2_-induced necrosis and camptothecin-induced apoptosis (data not shown), suggesting that selected compounds can induce autophagy as a cytoprotective mechanism. We next measured the capacity of compounds to activate autophagy following the conversion of cytosolic LC3 I to autophagosome associated LC3 II and found that LC3 II expression level was significantly increased by 1 μM SG6163F (>1.5 fold) and 3 μM rapamycin (>1.4 fold) treatment (Figure 4D). Next, RVNCs were transiently transfected with a GFP-LC3 plasmid, and localization of GFP-LC3 protein at autophagosomes was monitored by fluorescence 24 h post-transfection. Only treatments with 1 μM and 10 μM SG6163F and 10 μM digoxin were able to induce autophagosome formation, as shown in Figure 4E. 

**Figure 5:**
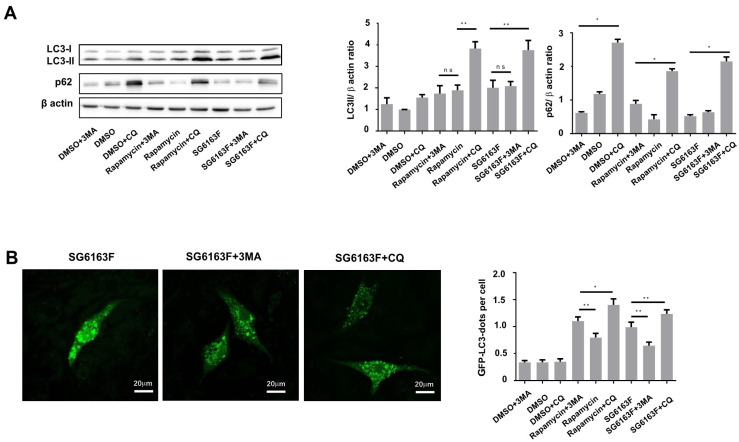
SG6163F stimulates autophagic flux in RNVCs. (**A**) Protein levels of LC3-I/II and p62 in RNVCs treated with 5 mM 3-methyladenine (3MA) and 20 μM chloroquine (CQ) and 10 μM SG6163F or 3μM rapamycin for 6 h were analyzed by Western blot. LC3II/β-actin and p62/β-actin ratios were determined and presented as fold change in comparison to DMSO. Data are presented as mean ± SEM with one-way ANOVA, Sidak’s multiple comparisons test. *, *p* < 0.05, **, *p* < 0.01. (**B**) Upon cell transfection by GFP-LC3 for 24 h and 6 h of cell treatment by SG6163F, 3-methyl adenine (MA), chloroquine (CQ), and rapamycin (not shown as image), GFP-LC3 redistribution to vacuoles (dots) was visualized by fluorescence microscopy and quantified by Image J. Experiments were repeated three times. Data are presented as mean ± SEM with one-way ANOVA, Sidak’s multiple comparisons test, *, *p* < 0.05, **, *p* < 0.01, ns, no significant.

We further measured the autophagic flux monitoring the accumulation of LC3 II and the ubiquitin-scaffold binding protein p62 after treatment with two autophagy inhibitors, 3-methyladenine (3MA) and chloroquine (CQ) (Figure 5). In the presence of CQ, but not 3MA, we observed an accumulation of LC3-II and p62 after cell treatment with SG6163F and rapamycin (Figure 5A) and an increase in GFP-LC3 puncta (Figure 5B). 

Altogether, these results reveal that all compounds require ATG5 and BECLIN1 to exert their cell death inhibitory activity, but only SG6163F stimulates the autophagic flux. 

### 3.3. Compounds Impact on Mitochondrial Network Structure and Dynamics

**Figure 6:**
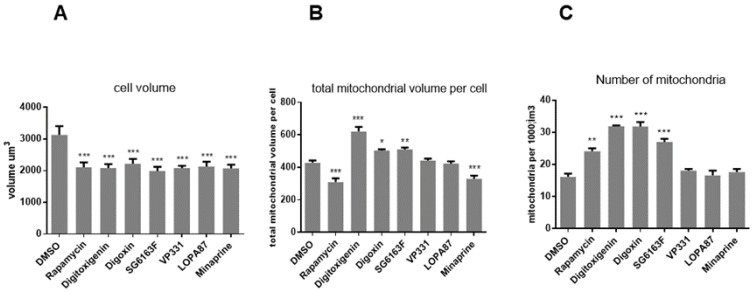
Effect of selected compounds on mitochondrial volume. RNVCs were treated with 1 μM compounds for 6 h and labeled with 4 μM calcein to determine the effect of compounds on the mitochondrial volume (**A**). Mitochondria were labeled with 200 nM Mitotracker to evaluate the total volume of mitochondria per cell (**B**) and quantify the number of individual mitochondria per cell (**C**). At least 150 cells were analyzed using a Leica confocal microscope and IMARIS software. Data are presented as mean ± SEM with one-way ANOVA, Sidak’s multiple comparisons test. *, *p* < 0.05, **, *p* < 0.01, ***, *p* < 0.001 vs. DMSO. Experiments were repeated three times.

**Figure 7:**
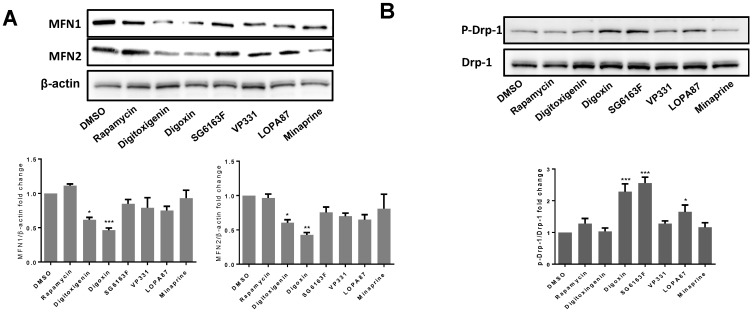
Effects of compounds on the expression of proteins of the mitochondria fusion/fission machinery. (**A**) Following RNVCs incubation with 1 μM of indicated compounds, expression was analyzed by Western blot. The intensities of MFN1 and MFN2 bands were normalized to β-actin. (**B**) Drp-1 and *p*-Drp-1 expressions in treated RNVCs were analyzed by Western blot and their ratio quantified. Experiments were repeated 3 times. Representative Western blot images and quantification of three independent experiments are presented as mean ± SEM with one-way ANOVA, Sidak’s multiple comparisons test. *, *p* < 0.05, **, *p* < 0.01, ***, *p* < 0.001 vs. DMSO.

We next verified the effects on mitochondria in cells treated with different compounds because these organelles play a major role in cardiomyocyte cell functioning [7,19,20]. Following RNVCs treatment by the compounds for 6 h, the mitochondria were labeled with 200 nM Mitotracker and the cells with 4 μM calcein-AM. The mitochondrial network was visualized by confocal microscopy, and the numbers of individual mitochondria were analyzed using the software IMARIS. While all compounds significantly decreased the cell volume compared to the vehicle (Figure 6A), digitoxigenin, digoxin, and SG6163F increased the number of mitochondria and the total mitochondrial volume per cell (Figure 6B,C). In contrast, VP331, LOPA87, and minaprine had no effect on the number of mitochondria, whereas 10 μM minaprine and 3 μM rapamycin decreased the total mitochondrial network volume (Figure 6B,C). Moreover, digitoxigenin and digoxin decreased the expression of MFN1 and MNF2 proteins, essential for mitochondrial fusion (Figure 7A) and digoxin and SG6163F stimulated fission as detected by phosphorylation of Drp-1 at Ser616 (Figure 7B), which suggests that treatment with these compounds influences mitochondrial dynamics and induces mitochondrial fission. 

**Figure 8:**
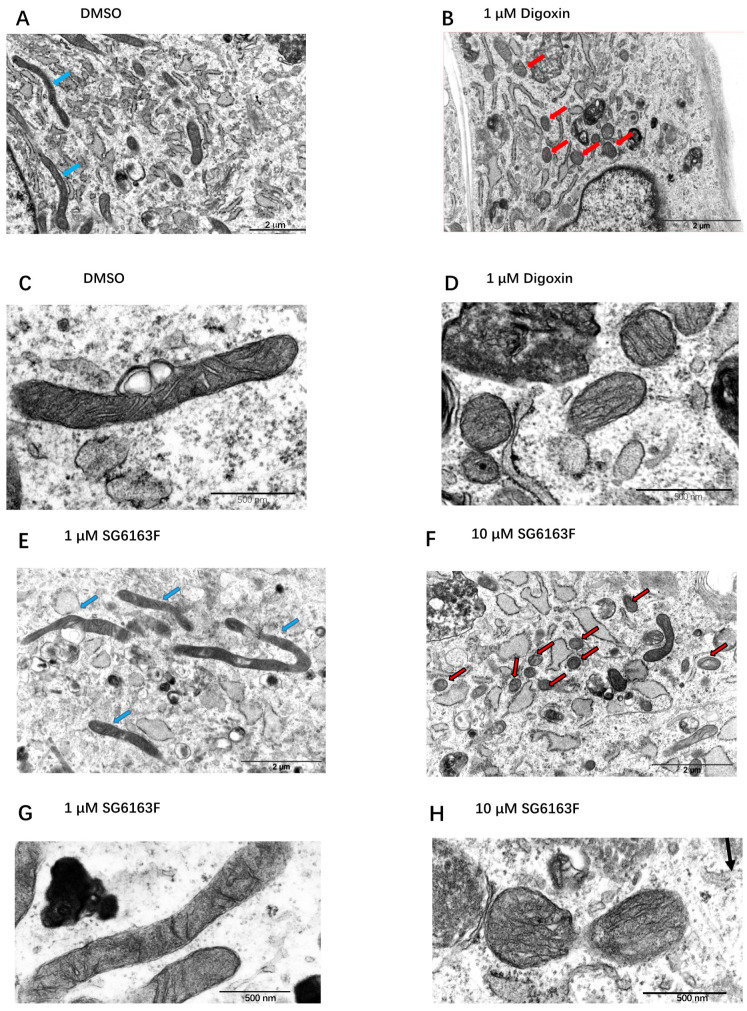
Mitochondrial morphology analysis in cells treated with SG6163F and digoxin by transmission electron microscopy (TEM). Cells were treated by 0.1% DMSO (**A**,**C**), 1 μM digoxin (**B**,**D**), 1 μM SG6163F (**E**,**G**), and 10 μM SG6163F (**F**,**H**), fixed by glutaraldehyde and analyzed by TEM. Blue arrows in (**A**,**E**) indicate the long and thin mitochondria, and the red arrows in (**B**,**F**) indicate short round mitochondria which suggest fission events.

In digoxin and SG6163F- treated cells, numerous short and round mitochondria can be observed compared to 0.1% DMSO-treated cells (controls), which have long and thin mitochondria (Figure 8). Thus, transmission electron microscopy confirms that mitochondria are smaller in H9c2 cells treated with 10 μM SG6163F and 1 μM digoxin in comparison to cells treated with DMSO or 1 μM SG6163F.

### 3.4. Metabolic Reprogramming in Cells Treated with Selected Compounds

**Figure 9:**
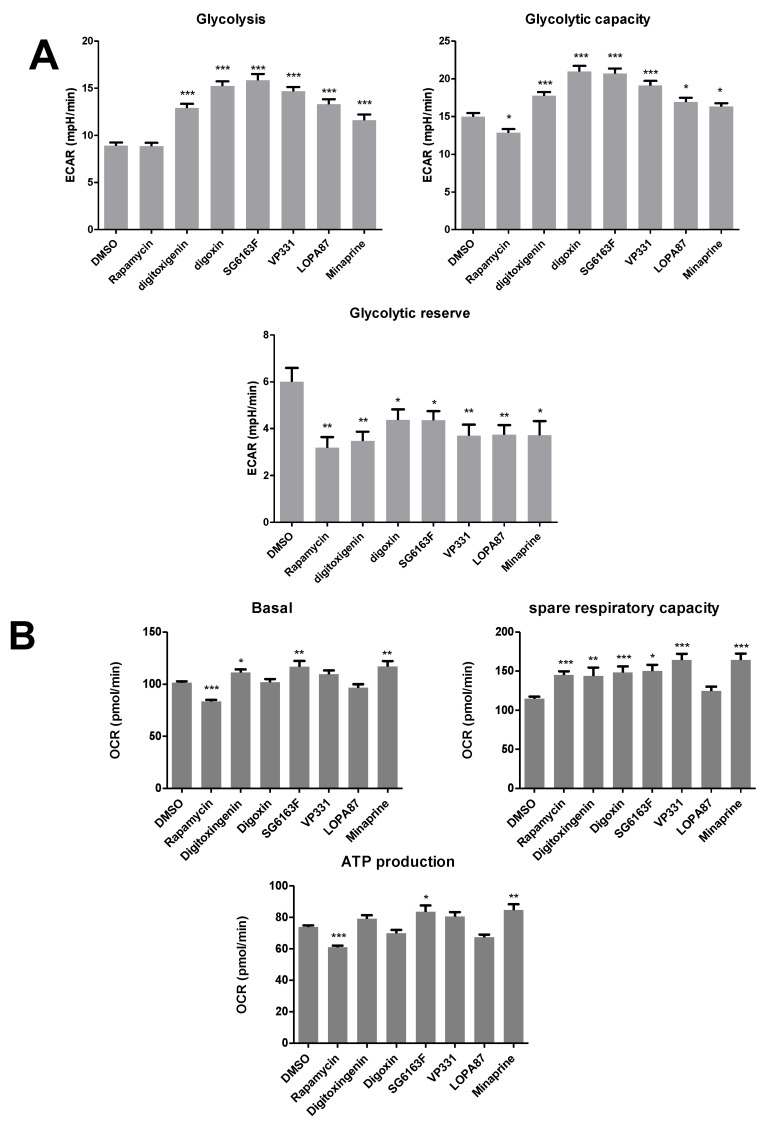

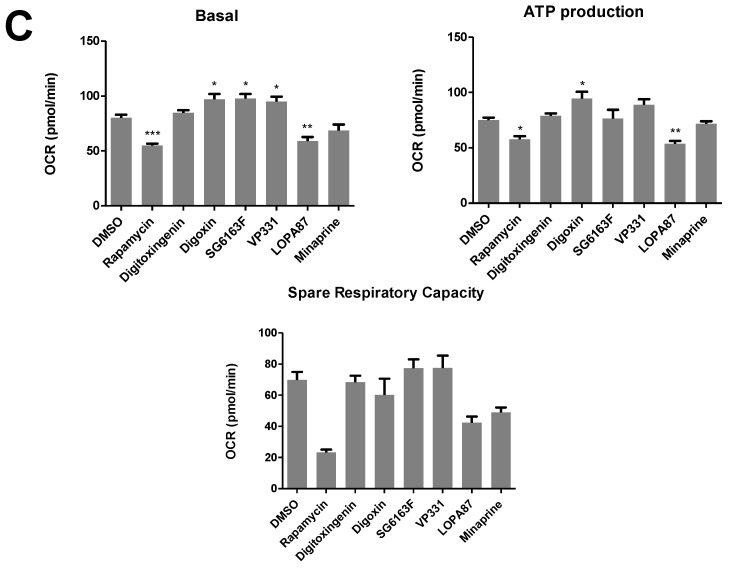
Metabolic reprogramming effects. (**A**) H9c2 cells were treated with indicated compounds for 6 h, and glycolytic function (**A**) and mitochondrial respiration (**B**) were measured by XFe96 Extracellular Flux Analyzer. (**C**) The oxidation of exogenous fatty acids was measured using the XF Palmitate-BSA FAO Substrate kit. OCR rates are expressed for 20,000 cells per well. Experiments were repeated three times. Data are presented as mean ± SEM with one-way ANOVA, Sidak’s multiple comparisons test. *, *p* < 0.05, **, *p* < 0.01, ***, *p* < 0.001 vs. DMSO.

To dissect the metabolic effects of compounds, we analyzed the energy metabolism of H9c2 cells in real time. We found that all compounds except rapamycin increased extracellular acidification suggesting an increase of ATP productions by anaerobic glycolysis (Figure 9A). Digitoxigenin and minaprine improved ATP production by oxidative phosphorylation (OXPHOS) using glucose and pyruvate, but not fatty acids as substrates (Figure 9B,C). VP331 Digoxin improved OXPHOS using fatty acids as substrate (Figure 9C) but not glucose (Figure 9B). SG6163F boosted OXPHOS, but rapamycin decreased it [21] (Figure 9B,C).

Finally, we evaluated ROS production by detecting anion superoxide in RVNCs by MitoSOX fluorescent probe following cell treatment by the compounds during 6 h. We found that rapamycin, digitoxigenin, VP331, LOPA87, and minaprine but not digoxin or SG6163F induced a local mitochondrial anion superoxide production in line with the observed activation of OXPHOS (Supplementary Figure S2).

## 4. Discussion

By high-throughput screening, we have identified new compounds capable of inhibiting cardiac apoptosis and necrosis and characterized their effects in H9c2 cells and in primary RNVCs. Among these compounds, digitoxigenin and digoxin (cardiac glycosides) and minaprine are molecules from Prestwick library, a commercial library of 1200 off-patent small molecules, 95% being approved drugs. Three other chemicals, SG6163F, VP331, and LOPA87, are new small molecules, which were synthesized in our laboratories [13,14].

Cardiac glycosides are natural molecules used in clinical medicine known for their antagonistic action on Na^+^,K^+^-ATPase. Cardiac glycosides have increased sensitivity in cancer cells [22] and have an ability to induce apoptosis [23], promote immunogenic cell death [24], and mediate autosis, a form of cell death resulting from excessive autophagy [25]. Here, we observed that digitoxigenin and digoxin promote cell death of A549 lung cancer cells (Figure 2) and have a pro-survival ATG5 and BECLIN-1-dependent autophagic activity in RNVCs (Figure 3). This is accompanied by a downregulation of BCL-XL and BAX, two members of the BCL-2 family, but no effect on the BCL-2 expression level was observed (Figure 3 and Figure 4). Importantly, because the disruption of interaction of BECLIN-1 and BCL-XL induces autophagy [26], our results are in line with the major role of BECLIN-1 in the heart, where changes in its expression affect functions and survival of cardiomyocytes [27].

Our results show that pharmacological manipulation of autophagy can be instrumental for protection from H_2_O_2_-induced oxidative alterations and DNA-damage events induced by camptothecin. Indeed, all compounds required BECLIN-1 and ATG5 to protect primary cardiomyocytes from cell death, as shown by the downregulation of these autophagy activators upon siRNA treatment (Figure 4). However, only SG6163F and digoxin treatment were shown to involve autophagosome formation (Figure 4D,E).

Treatments of RNVCs by compounds revealed that digitoxigenin, digoxin, and SG6163F modulate mitochondrial dynamics and/or biogenesis by increasing mitochondrial mass and number of mitochondria accompanied by a decrease of cell volume (Figure 6). In addition, cardiac glycosides, but not SG6163F, decreased the expression level of mitofusins MFN1 and MFN2, while digoxin and SG6163F activated organelle fission as revealed by phosphorylation of DRP1 on Ser 616 (Figure 7). In addition, digitoxigenin, digoxin, and SG6163F stimulated ATP production by anaerobic glycolysis. Digoxin and SG6163F boosted glucose and pyruvate-fueled OXPHOS (Figure 8), while digitoxigenin stimulated fatty acid-fueled OXPHOS. 

In summary, digitoxigenin, digoxin, and SG6163F protected cardiomyocytes by decreasing the expression of pro-apoptotic protein BAX, affecting autophagy, increasing mitochondrial mass, and boosting ATP production by improving aerobic and anaerobic metabolism. However, the three molecules effects differ in BCL-XL, MFN, DRP-1/DRP1-*p* expression regulation, ROS production, and toxicity for cancer cell line A549. In contrast, VP331, LOPA87, and minaprine had no effect on BCL-2 family expression (Figure 3) and required ATG5 and BECLIN-1 (Figure 4). Mitochondrial ROS increase was observed following RNVCs treatments by VP331, LOPA87, and minaprine, which could be due to OXPHOS stimulation (Supplementary Figure S2). These molecules also boosted ATP production by anaerobic glycolysis. 

To conclude, we conducted a robust high-throughput screening to search for cell death inhibitors. These assays are complementary to the previous low-throughput screens [28,29,30,31]. Our screening identified six inhibitors of cardiac cell death, which act through autophagy and metabolism reprogramming. These compounds have promising cardioprotective activities and, thus, might be useful in clinics for repositioning purposes or as new drug candidates. Since there is a high interconnection between metabolism, cell death, and malignancy [32,33], it was important to check the effect of our compounds on cancer cell proliferation. None of the six compounds favored cell proliferation in A549 lung cancer cells or led to the overexpression of oncogenic proteins BCL-2 and BCL-XL (Figure 2 and Figure 3). In addition, all molecules showed no cytotoxicity for RNVCs and H9c2 cells (Figure 2). These are particularly interesting results in the perspective of the use of compounds in anticancer combination therapy. 

As anticipated from chemical structures, compounds were rapidly metabolized in vitro mouse microsomes or showed poor solubility (data not shown), which might hamper preclinical studies in animals [34]. Therefore, if digitoxigenin, digoxin, and minaprine as approved FDA molecules could be repositioned and enter rapidly in preclinical studies in combination with radiation or chemotherapeutic agents, the three new compounds, SG6163F, VP331, and LOPA87, may require chemical optimization for further therapeutic development.

## Supplementary Materials

The following supporting information can be downloaded at: https://www.mdpi.com/article/10.3390/cells11030474/s1, Figure S1: Selected compounds inhibition of H_2_O_2_ induced-necrosis in RNVCs and H9c2; Figure S2: Effects of compounds on mitochondrial ROS production.

## Abbreviations

LDH	Lactate dehydrogenase
SD	Standard deviation
3-MA	3-methyladenine
CQ	Chloroquine
