# Supplementary material for: Identification of Small Molecules Inhibiting Cardiomyocyte Necrosis and Apoptosis by Autophagy Induction and Metabolism Reprogramming
Source: Cells. 2022 Jan 29;11(3):474. doi: 10.3390/cells11030474 (PMC8834338; doi:10.3390/cells11030474)
Supplement: Supplementary file 1 [file cells-11-00474-s001.zip › cells-1489859-supplementary.pdf]

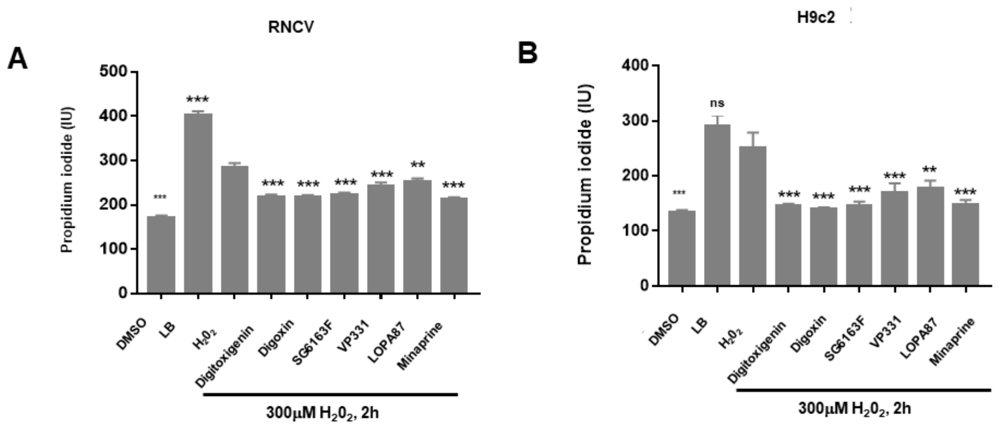

Figure Supp 1

Figure S1. Selected compounds inhibition of H_2O_2 induced-necrosis in RNVCs and H9c2. (**A**) RNVCs and (**B**) H9c2 cell membrane permeabilisation was measured following propidium iodide labelling by spectrofluorimetry. Data are presented as mean ± SEM with one-way ANOVA, Sidak's multiple comparisons test. *, $p < 0.05$, **, $p < 0.01$, ***, $p < 0.001$ vs DMSO.

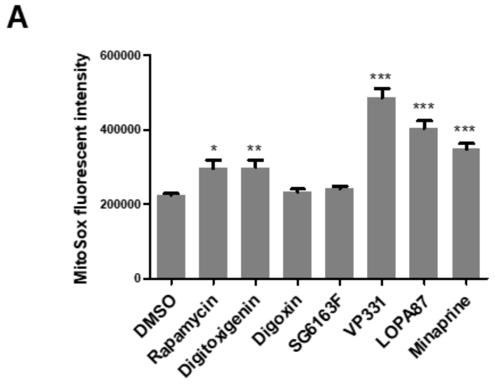

Figure S2. Effects of compounds on mitochondrial ROS production. RNVCs were treated with 0.1% DMSO, 3 μM rapamycin, 1 μM of digitoxigenin, digoxin, minaprine, VP331, LOPA87 and SG6163F for 6 h, then ROS production was detected by 5 μM MitoSOX fluorescent probe. Fluorescence was captured by Leica confocal microscope and quantification of mitochondrial fluorescent intensity was evaluated by Image J. Data are presented as mean ± SEM with one-way ANOVA, Sidak's multiple comparisons test. *, $p < 0.05$, **, $p < 0.01$, ***, $p < 0.001$ vs DMSO.
